# Pharmacodynamic Functions of Synthetic Derivatives for Treatment of Methicillin-Resistant *Staphylococcus aureus* (MRSA) and *Mycobacterium tuberculosis*

**DOI:** 10.3389/fmicb.2020.551189

**Published:** 2020-11-27

**Authors:** Mojdeh Dinarvand, Malcolm P. Spain, Fatemeh Vafaee

**Affiliations:** ^1^School of Chemistry, Faculty of Science, The University of Sydney, Sydney, NSW, Australia; ^2^Department of Infectious Diseases and Immunology, Faculty of Medicine and Health, The University of Sydney, Sydney, NSW, Australia; ^3^School of Biotechnology and Biomolecular Sciences, Faculty of Science, University of New South Wales, Sydney, NSW, Australia

**Keywords:** marine natural products, antibiotic resistance (AMR), time-kill studies, checkerboard assay, methicillin-resistant *Staphylococcus aureus*, *Mycobacterium tuberculosis*

## Abstract

Drug resistant bacteria have emerged, so robust methods are needed to evaluate combined activities of known antibiotics as well as new synthetic compounds as novel antimicrobial agents to treatment efficacy in severe bacterial infections. Marine natural products (MNPs) have become new strong leads in the drug discovery endeavor and an effective alternative to control infections. Herein, we report the bioassay guided fractionation of marine extracts from the sponges *Lendenfeldia*, *Ircinia*, and *Dysidea* that led us to identify novel compounds with antimicrobial properties. Chemical synthesis of predicted compounds and their analogs has confirmed that the proposed structures may encode novel chemical structures with promising antimicrobial activity against the medically important pathogens. Several of the synthetic analogs exhibited potent and broad spectrum *in vitro* antibacterial activity, especially against the Methicillin-resistant *Staphylococcus aureus* (MRSA) (MICs to 12.5 μM), *Mycobacterium tuberculosis* (MICs to 0.02 μM), *uropathogenic Escherichia coli* (MIC o 6.2 μM), and *Pseudomonas aeruginosa* (MIC to 3.1 μM). Checkerboard assay (CA) and time-kill studies (TKS) experiments analyzed with the a pharmacodynamic model, have potentials for *in vitro* evaluation of new and existing antimicrobials. In this study, CA and TKS were used to identify the potential benefits of an antibiotic combination (i.e., synthetic compounds, vancomycin, and rifampicin) for the treatment of MRSA and *M. tuberculosis* infections. CA experiments indicated that the association of compounds 1a and 2a with vancomycin and compound 3 with rifampicin combination have a synergistic effect against a MRSA and *M. tuberculosis* infections, respectively. Furthermore, the analysis of TKS uncovered bactericidal and time-dependent properties of the synthetic compounds that may be due to variations in hydrophobicity and mechanisms of action of the molecules tested. The results of cross-referencing antimicrobial activity, and toxicity, CA, and Time-Kill experiments establish that these synthetic compounds are promising potential leads, with a favorable therapeutic index for antimicrobial drug development.

## Introduction

The first isolation of methicillin-resistant *Staphylococcus aureus* (MRSA) and *Mycobacterium tuberculosis* was reported in 1882 and 1961, respectively (Enright et al., 2002; Cambau and Drancourt, 2014). The increasing prevalence of antibiotic resistance and lack of clinically approved treatments has made these infections a new challenge for infection control teams worldwide (Enright et al., 2002; Zaman, 2010; Diallo et al., 2018; Yang et al., 2018). This determined existing antibiotic therapies require substantial improvements in efficacy, in order to overcome severe chronic bacterial infections (Broussou et al., 2019; Dinarvand and Peter Spain, 2020).

Marine ecosystems have long been a rich source of bioactive natural products, in the search for interesting molecules and novel therapeutic agents (Molinski et al., 2009; Hughes and Fenical, 2010; Indraningrat et al., 2016; Mehbub et al., 2016; Schroeder et al., 2018; El-Demerdash et al., 2019) over the last 70 years (Newman and Cragg, 2016; Blunt et al., 2017, 2018; Pye et al., 2017). The preclinical pharmacology of seventy-five compounds isolated from marine organisms was reported to have biological activities (Mayer et al., 2017).

The most prolific marine organisms are sponges (Indraningrat et al., 2016), and the oldest metazoans on earth belong the phylum *Porifera* (Matobole et al., 2017). The Demospongiae, being most abundant class of Porifera, represent 83% of described species (Van Soest et al., 2012; Matobole et al., 2017) and have the largest number of bioactive compounds (El-Damhougy et al., 2017).

Member of the genus *Lendenfeldia* are a known source of sulfated sterols (Radwan et al., 2007). The *Lendenfeldia* species metabolites have anti-HIV, anti-tumor (Liu et al., 2008), anti-inflammatory, and antifouling (Sera et al., 1999) activities, but they are believed to lack antimicrobial activity (Radwan et al., 2007). Secondary metabolites of the genus *Ircinia* and *Dysidea* have been well studied for antibacterial activity (Thakur and Anil, 2000; Mohamed et al., 2008; Belma Konuklugil, 2015; El-Damhougy et al., 2017).

In fact, the first ‘drugs from the sea’ were only approved in the early 2000s. They included: the cone snail peptide ziconotide (ω-conotoxin MVIIA) in 2004 to alleviate chronic pain (McGivern, 2007) and sea squirt metabolite trabectedin in 2007 for treatment of soft-tissue sarcoma (Gordon et al., 2016). Marine natural products (MNPs) have also displayed exceptional potential as anticancer therapeutics (El-Damhougy et al., 2017). Interest in MNPs has continued to grow since (Newman and Cragg, 2016; Blunt et al., 2017, 2018), spurred in part by the spread of antimicrobial resistant pathogens and the need for new drugs to combat them (Indraningrat et al., 2016). In search of novel antimicrobial agents, we previously elucidated five active component structures using high resolution and tandem mass spectrometry (MS) which resulted in a series of potential structures for new, bioactive amine natural products (Dinarvand and Peter Spain, 2020). Generally, successful antimicrobial therapy depends on complex interactions between different factors like the infecting agent, host and the dose-response (Mueller et al., 2004).

To validate the proposed structures and explore the potential of this compound class more broadly, analogs of five general structures were synthesized and evaluated as potential antimicrobial agents against the medically important microorganisms: MRSA, *M. tuberculosis*, *UroPathogenic Escherichia coli* (UPEC), and *Pseudomonas aeruginosa*.

Checkerboard assay (CA) (Odds, 2003) was used to measure interaction between inhibitory agents categorized into synergy, indifference or antagonism. Furthermore, time-kill studies (TKS) (Broussou et al., 2019) experiment performed to study dynamic interpretation of drug-bacteria interactions. Also, combining existing antimicrobials has been successfully applied to optimize dosing strategies, extend the spectrum, minimize the emergence of resistance, enhance the bactericidal activity, manage an infection and prevent treatment failures (Foerster et al., 2016; Broussou et al., 2019).

## Materials and Methods

### General

Chemical reagents were purchased from BDH Chemicals and Sigma Aldrich (Castle Hill, Sydney, Australia) and used as supplied unless otherwise indicated.

### Natural Product Library

Natural product extracts were provided by the Australian Institute of Marine Science (AIMS), Townsville, Queensland as part of the AIMS Bioresources Library (Evans-Illidge et al., 2013), via the Queensland Compound Library (Simpson and Poulsen, 2014) (now called Compounds Australia (Compounds Australia, 2018)). Crude extracts had been partially fractionated by AIMS/QCL to generate a library of 1,434 samples, supplied in DMSO (100%) solution and stored at −80°C. Original concentrations as provided were 5 mg mL^–1^. Stock solutions were made by diluting these samples by a factor of 1:10 in dH_2_O and stored at −80°C.

### Bacterial Strains

*Pseudomonas aeruginosa* PAO1 was provided by Dr. Jim Manos, University of Sydney. *E. coli* EC958 was a kind gift of Professor Mark Schembri, University of Queensland. Mycobacterial strains (*M. tuberculosis* H37Rv, *M. bovis* BCG Pasteur, *M. smegmatis* mc2155) are laboratory stocks from the Triccas group (University of Sydney). The methicillin-resistant *Staphylococcus aureus* (MRSA) strain was clinical isolation provided by Dr. John Merlino (Concord Hospital NSW, Australia).

### Bacterial Inhibition Assays

For screening of the AIMS library, each test sample (10 μL) was dispensed into a separate well of a 96 well microtiter plates (final sample concentration 0.5 mg mL^–1^) using sterile dH_2_O. For determination of minimum inhibitory concentrations (MIC), extracts (250 to 0.5 μg mL^–1^) or synthesized compounds (100 to 0.0002 μM) were serially diluted in microtiter plates. Bacterial suspension (90 μL, OD 600 nm 0.001) was added to each well and plates were incubated at 37°C for either 18 h (MRSA, *P. aeruginosa* PAO1, *E. coli* EC958) or 7 days (*M. tuberculosis* H37Rv) as described previously (Harrison et al., 2017; Irene et al., 2019; Larcombe et al., 2019; Mouton et al., 2019). Resazurin (10 μL; 0.05% w/v) was added and plates were incubated for 3 h or 24 h (*M. tuberculosis*) at 37°C. The inhibitory activity was calculated by visual determination of color change within wells or detection of fluorescence at 590 nm using a FLUOstar Omega microplate reader (BMG Labtech, Germany). Percentage survival was calculated in comparison to the average of untreated control wells after normalizing for background readings.

### Evaluating Toxicity of AIMS Extract Library

Human alveolar epithelial cells (A549), (Giard et al., 1973), Madin-Darby canine kidney epithelial cells (MDCK), (Gaush et al., 1966), human leukemia cells (THP-1) (Tsuchiya et al., 1980), human hepatocellular carcinoma cells (Hep-G2), (Aden et al., 1979), and human embryonic kidney cells 293 (HEK293) (Graham et al., 1977) were grown and differentiated in complete RPMI (Roswell Park Memorial Institute Medium) and DMEM (Dulbecco’s Modified Eagle’s medium) tissue culture media (RPMIc and DMEMc). To determine toxicity of the AIMS extract library, 2 × 105 of each cell type were added to a 96-well plate and left for 48 h at 37°C to adhere. Extract samples at a final concentration of 0.5 mg mL^–1^ were added to the wells, then incubated for 7 days in a humidified 5% CO_2_ incubator at 37°C. Then resazurin (10 μL of 0.05% w/v) was added, and after 4 h, fluorescence measured as described previously. Cell viability was calculated as percentage fluorescence relative to untreated cells.

### Bacterial Growth Conditions and Curves

Bacterial growth was monitored over a time-course of 60 h (0, 2, 4, 6, 8, 10, 12, 20, 22, 24, 26, 28, 30, 32, 34, 40, 44, 48, and 60 h) and 7 days (24, 48, 72, 96, 120, 144, and 168 h) for A MRSA and *M. tuberculosis* H37Ra, respectively. For every sampled time point harvest, 1 mL aliquot of the sample was isolated and viable counts were determined. Growth curves were analyzed by plotting the log CFU/ml against the time using previously developed R scripts (version 3.2.0) (Foerster et al., 2016). Only lag, log, and stationary phases were included in the analysis while the decline phase was excluded.

### Antibiotics

Vancomycin and Rifampicin powders were dissolved in DMSO to prepare antibiotic stock solutions in 2and 50 mM concentrations, respectively. The stocks were stored at −20°C for less than 1 month. Antibiotic solutions were thawed and diluted to the desired concentrations just before use.

### Determination of the Minimum Bactericidal Concentration (MBC)

Bacterial solutions (100 μL) were taken from the two lowest concentrations of the well exhibiting invisible growth (clear well) and sub-cultured onto sterile agar plates. The MRSA and *M. tuberculosis* H37Ra plates were incubated at 37°C for 18 h and 7 days, respectively, and then examined for bacterial growth. MBC was taken as the concentration of antimicrobial agents that did not exhibit any bacterial growth on the freshly inoculated agar plates. Whole experiments were performed in triplicate, and each experiment was repeated a minimum of three times.

### Checkerboard Assays (CA)

Referring to the MICs of six synthetic compounds, CA were designed to determine their fractional inhibitory concentrations (FICs) in combinations against MRSA and *M. tuberculosis* H37Rv. The tests were performed on 96-well plates according to previous methods (Odds, 2003; Jacqueline et al., 2005, 2006; Xu et al., 2018). Briefly, aliquots (10 μL) of four-fold dilutions of the drug solutions (vancomycin and rifampicin) were added to each plate, such that the vancomycin concentrations ranged from 100 to 25 μg mL^–1^, and rifampicin concentrations from 4.0 to 0.25 μm were added to each well in the plates. Then solutions of the synthetic samples (10 μL) were added to plates, in order to yield final drug concentrations spanning their MICs. Finally, bacterial stock suspensions (80 μL) were added to all wells with MIC. In addition, bacteria were quantified in each well to determine the MBC. FICs were calculated using below equation:

F⁢I⁢C⁢(X+Y)=[M⁢I⁢C⁢o⁢f⁢c⁢o⁢m⁢p⁢o⁢u⁢n⁢d⁢X⁢i⁢n⁢c⁢o⁢m⁢b⁢i⁢n⁢a⁢t⁢i⁢o⁢n⁢w⁢i⁢t⁢h⁢Y][M⁢I⁢C⁢o⁢f⁢X⁢a⁢l⁢o⁢n⁢e]-1

The ratio of FIC to MIC is referred to as the FIC index (Odds, 2003; Cikman et al., 2015; Guy et al., 2017). To evaluate interaction profiles, the fractional inhibitory index (ΣFIC) was calculated as a sum of both FIC drug X (antimycobacterial drug) and FIC drug Y (synthetic amine) where ΣFICs is smaller than 0.25 designated synergistic activity, 0.25–4 indifferent or additive activity and >4 indicate antagonism (Odds, 2003; Cikman et al., 2015; Guy et al., 2017).

### sTKS (Constant Antibiotic Concentrations)

Time-kill experiments were performed by culturing MRSA and *M. tuberculosis* in liquid medium in the presence of six synthesized sample concentrations in doubling dilutions ranging from 8 to 0.001 × MIC and 2 × MIC for both bacteria, respectively. Growth curves were initially generated to confirm that bacteria would reach a stable early- to mid-log phase after 2 and 48 h of pre-incubation in antimicrobial-free both medium, respectively. Samples were taken in different incubation times after drug exposure at 0, 2, 4, 6, 8, and 24 h for MRSA and 24, 48 h, and 4 and 6 days for *M. tuberculosis* and then plated onto agar plates for CFU determination. The experiments were carried out in duplicate and repeated three times.

### Synthesis

#### *N,N*-Dioctyl-3-Methylaniline 1a

To a solution of 1-bromooctane 6 (6.3 mL, 36.6 mmol) in acetonitrile (50 mL) was added potassium carbonate (25.3 g, 183 mmol), KI (6.08 g, 36.6 mmol), and *m*-toluidine 7 (1.96 mL, 18.3mmol) stirred at 60°C overnight. The suspension was filtered and washed with acetonitrile (3 × 50 mL), then concentrated by rotary evaporation to yield the crude product (1.50 g). Purification by automated column chromatography (100 g cartridge, 100% petroleum benzine over 12 CV) then by preparative TLC (10% ethyl acetate:petroleum benzine) afforded the pure compound 1a as a yellow oil (0.46 g, 8%). 1H NMR (500 MHz, CDCl3): δ 7.13–7.07 (m, 1 H), 6.50–6.44 (m, 3 H), 3.28–3.22 (m, 4 H), 2.32 (s, 3 H), 1.64–1.53 (m, 4 H), 1.38–1.24 (m, 20 H), 0.94–0.87 (m, 6 H); 13C NMR (125 MHz, CDCl3): δ 148.3, 138.7, 129.0, 116.0, 112.4, 109.0, 51.0, 31.8, 29.5, 29.3, 27.3, 27.2, 22.7, 22.0, and 14.1; LRMS (ESI+): m/z 332.33 [M + H]+, 100%; HRMS (ESI): m/z calculated for [C23H42N]+, [M + H]+ 332.3317, found 332.33054.

#### *N,N*-Dioctyl-4-Methylaniline 1b

To a solution of 1-bromooctane 6 (6.3 mL, 36.6 mmol) in acetonitrile (50 mL) was added potassium carbonate (25.3 g, 183 mmol), KI (6.08 g, 36.6 mmol), and *p*-toluidine 8 (1.96 g, 18.3 mmol) stirred at 60°C overnight. The suspension was filtered then concentrated by rotary evaporation to yield the crude product (1.65 g). Purification by automated column chromatography (100 g cartridge, 100% petroleum benzine over 4 CV, ramping to 100% ethyl acetate over 4 CV) gave the product 1b as a yellow oil (0.39 g, 6%). 1H NMR (500 MHz, CDCl3): δ 7.02 (d, *J* = 8.2 Hz, 2 H), 6.68–6.47 (m, 2 H), 3.33–3.10 (m, 4 H), 2.25 (s, 3 H), 1.58–1.52 (m, 4 H), 1.40–1.20 (m, 20 H), 0.95–0.82 (m, 6 H); 13C NMR (125 MHz, CDCl3): δ 146.2, 129.7, 124.3, 112.2, 51.3, 31.9, 29.5, 29.4, 27.3, 27.2, 22.7, 20.1, and 14.1; LRMS (ESI+): m/z 332.33 [M + H]+, 100%; HRMS (ESI): m/z calculated for [C23H42N]+ [M + H]+ 332.3317, found 332.33095.

#### *N*-Benzyl-*N*-Octyloctan-1Amine 2a

To a solution of 1-bromooctane 6 (6.3 mL, 36.6 mmol) in acetonitrile (50 mL) were added potassium carbonate (25.3 g, 183 mmol) and benzylamine 9 (2 mL, 18 mmol) then stirred at 60°C overnight. The mixture, a clear colorless solution, was concentrated using the rotary evaporator, to give a white solid. The solid was triturated with DCM (1 × 50 mL, 1 × 25 mL) and the DCM solution concentrated on the rotary evaporator to yield yellow oil (5.1 g). Purification by automated column chromatography (100 g cartridge, 0–40% ethyl acetate (EtOAc) in petroleum benzine over 10 CV) yielded *N*-benzyl-*N*-octyloctan-1-amine 2a as a colorless oil (1.15 g, 19%). 1H NMR (500 MHz, CDCl3): δ 7.36–7.29 (m, 4H), 7.26–7.21 (m, 1H), 3.56 (s, 2H), 2.47–2.35 (m, 4H), 1.54–1.41 (m, 4H), 1.36–1.21 (m, 20H), 0.90 (t, *J* = 7.0 Hz, 6H); 13C NMR (125 MHz, CDCl3): δ 140.3, 128.8, 128.0, 126.6, 58.6, 53.8, 31.9, 29.6, 29.3, 27.5, 27.0, 22.7, and 14.1; LRMS (ESI+): m/z 332.33 [M + H]+, 100%; HRMS (ESI): m/z calculated for [C23H42N]+ [M + H]+ 332.3317, found 332.3306.

#### *N,N-*Dihexyldecan-1-Amine 3

To a solution of 1-iododecane 10 (2.75 mL, 12.9 mmol) in acetonitrile (60 mL) was added potassium carbonate (16.5 g, 129 mmol) and dihexylamine 11 (3 mL, 12.9 mmol), then the mixture was stirred at reflux overnight. The suspension was filtered to remove K_2_CO_3_ and washed with acetonitrile (3 × 50 mL), then concentrated by rotary evaporation to yield the crude product (7.8 g). The crude product was purified by automated column chromatography (100 g cartridge, 100% petroleum benzene 2CV, then 0–60% ethyl acetate in petroleum benzene over 10 CV) to yield the product as an oil (0.89 g, 21%). 1H NMR (500 MHz, CDCl3): δ 2.41–2.37 (m, 6 H), 1.38–1.46 (m, 6 H), 1.35–1.21 (m, 28 H), 0.91–0.86 (m, 9 H); 13C NMR (125 MHz, CDCl3): δ 54.2,54.2, 31.9, 31.9, 29.7, 29.6, 29.6, 29.3, 27.7, 27.4, 26.9, 22.7, 14.1, and 14.1; LRMS (ESI+): m/z 326.41 [M + H]+, 100%; HRMS (ESI): m/z calculated for [C22H48N]+ [M + H]+ 326.3787, found 326.3776.

#### *N,N,N*-Trioctylammonium Chloride 13

To a solution of *N,N,N*-trioctylamine 12 (1.0 g, 2.80 mmol) in 1,4-dioxane (1.0 mL) was added 4 M HCl in dioxane (2.80 mL, 11.2 mmol) in an ice bath. Instantaneously a precipitate formed and after 5 min this was collected by vacuum filtration to yield a white solid (1.08 g, 99% yield). 1H NMR (500 MHz, CDCl3) δ 11.40 (br s, 1 H), 2.90 (td, *J* = 4.7, 12.5 Hz, 6 H), 1.74–1.66 (m, 6 H), 1.29–1.13 (m, 30 H), 0.79 (t, *J* = 7.0 Hz, 9 H); 13C NMR (125 MHz, CDCl3) δ 52.2, 31.4, 28.8, 28.7, 26.6, 23.0, 22.3, and 13.8.

#### *N*-(But-3-yn-1-yl)-*N*-Octyloctan-1-Amine 16

To a flask charged with potassium carbonate (4.4 g, 31.8 mmol) and potassium iodide (880 mg, 5.30 mmol) was added acetonitrile (72 mL) followed by dioctylamine 14 (8 mL, 26.5 mmol) and 4-bromo-1-butyne 15 (2.74 mL, 29.2 mmol). The suspension was stirred at 60°C for 18 h, then filtered and washed with acetonitrile (3 × 50 mL), and concentrated by rotary evaporation to yield the crude product. This was purified by automated column chromatography (100 g cartridge, 0%–15% ethyl acetate in petroleum benzine over 10 CV) to yield the product as a yellow oil (0.15 g, 2%). 1H NMR (500 MHz, CDCl3): δ 2.64–2.57 (m, 2 H), 2.39–2.31 (m, 4 H), 2.23 (dt, *J* = 2.7, 7.6 Hz, 2 H), 1.88 (t, *J* = 2.6 Hz, 1 H), 1.41–1.31 (m, 4 H), 1.27–1.14 (m, 20 H), 0.81 (t, *J* = 7.0 Hz, 6 H); 13C NMR (125 MHz, CDCl3): δ 83.3, 68.7, 54.0, 52.7, 31.8, 29.6, 29.3, 27.6, 27.2, 22.6, 16.7, and 14.1; LRMS (ESI+): m/z 294.29 [M + H]+, 100%; HRMS (ESI): m/z calculated for C20H40N+ [MH]+ 294.3161, found 294.3157.

#### 6-(4-(2-(Dioctylamino) Ethyl)-1*H*-1,2,3-Triazol-1-yl)-2-Ethyl-1*H*-Benzo[de]isoquinoline-1,3(2H)-Dione 18

To a solution of 16 (400 mg, 1.24 mmol) and 6-azido-2-ethyl-1H-benzo[de]isoquinoline-1,3(2H)-dione 17 (365 mg, 1.61 mmol) in tert-butanol: water (12.4 mL) were added copper sulfate hexahydrate (31.0 mg, 0.12 mmol) and ascorbic acid sodium salt (73.8 mg, 0.37 mmol) then the solution was stirred at 60°C overnight. Precipitants were removed by filtration and washed with acetonitrile (3 × 50 mL), then the filtrate was concentrated by rotary evaporation and purified by automated column chromotography (100 g cartridge, 0–20% methanol in dichloromethane over 8 CV). Purified fractions were re-purified by automated reversed phase chromotography (30 g cartridge C18 silica (Biotage), 0–90% acetonitrile in water over 17 CV) and concentrated to yield the product as a yellow solid (10 mg, 1%). 1H NMR (500 MHz, CDCl3): δ 8.73 (d, *J* = 7.6 Hz, 2 H), 8.21 (dd, *J* = 0.9, 8.5 Hz, 1 H), 8.09 (s, 1 H), 7.89–7.81 (m, 2 H), 4.29 (q, *J* = 7.1 Hz, 2 H), 3.67–3.56 (m, 2 H), 3.49–3.37 (m, 2 H), 3.25–3.06 (m, 4 H), 1.87–1.70 (m, 4 H), 1.38 (t, *J* = 7.0 Hz, 3 H), 1.34–1.23 (m, 10 H), 0.89 (t, *J* = 7.0 Hz, 6 H); 13C NMR (126 MHz, CDCl3): δ 162.4, 161.9, 142.1, 136.8, 131.2, 129.6, 128.1 (128.09), 128.1 (128.08), 127.7, 125.4, 124.1, 123.2, 122.6, 122.1, 51.6, 51.3, 34.8, 30.6, 28.7, 28.0, 25.7, 22.0, 21.5, 20.1, 13.0, and 12.3; LRMS (ESI+): m/z 560.38 [M + H]+, 100%; HRMS (ESI): m/z calculated for C34H50N5O2+ [MH]+ 560.3965, found 560.3950.

## Results and Discussion

### Identification of Active Marine Extracts

To identify marine samples with biological activity, 1,434 compounds from the AIMS Bioresources Library (Evans-Illidge et al., 2013; Simpson and Poulsen, 2014; Compounds Australia, 2018) were screened against MRSA in a resazurin cell viability assay. Twenty-three compounds from extracts and fractions derived from the phyla Porifera (90%), Echinodermata (5%), and Chordata (5%) inhibited of MRSA growth by greater than 50% compared to non-treated controls (Supplementary Table S1). MICs for promising samples were determined (Supplementary Table S1). The five most active samples showed MICs at 31.3 μg mL^–1^ (all Porifera samples), while another four samples returned MICs of 62.5 μg mL^–1^ (also all Porifera). Cytotoxicity screens against HepG2, HEK 293, A549 and THP-1 cell lines were performed to define the cytotoxicity profile of the most active samples (Supplementary Table S2). Pleasingly, all the samples most active against MRSA were also non-toxic to the cell lines tested.

### Synthesis of Bioactive Compounds From Marine Extracts

**Scheme 1:**
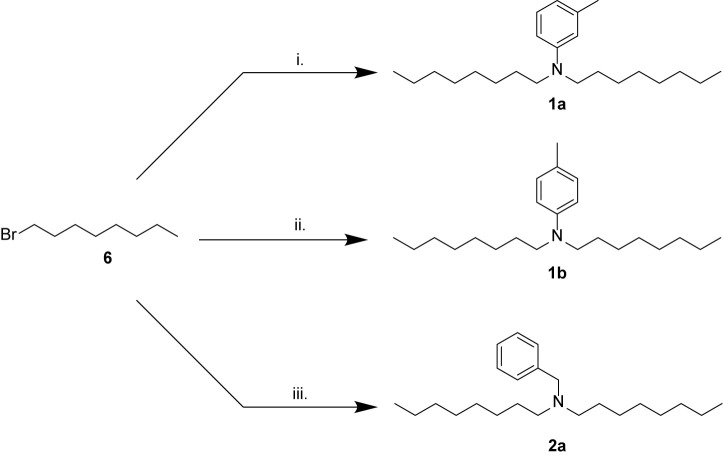
Synthesis of amines related to natural products 1 and 2. (i) *m*-toluidine 7, K2CO3, KI, MeCN, 60°C, overnight, 8%; (ii) *p*-toluidine 8, K2CO3, KI, MeCN, 60°C, overnight, 6%; (iii) benzylamine 9, K2CO3, MeCN, 82°C, overnight, 19%.

**Scheme 2:**
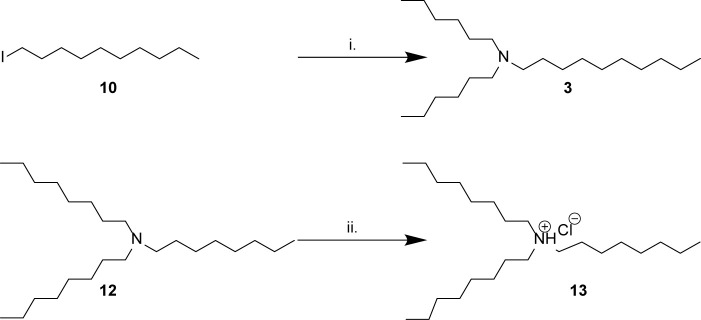
Synthesis of amines related to natural products 3–5. (i) *N,N*-dihexylamine 11, K2CO3, MeCN, 82°C, overnight, 21%; (ii) HCl in 1,4-dioxane, 5 min, 99%.

Five of the six bioactive components (Table 1) were isolated and characterized from extracts as a reported previously (Dinarvand and Peter Spain, 2020). To validate the structures proposed for the natural products, and to explore the potential of these a compounds as bioactive agents, a series of tertiary amine derivatives of compounds 1–5 (Dinarvand and Peter Spain, 2020) were synthesized from 1-bromooctane 6, *m*-toluidine 7, *p*-toluidine 8, benzyl amine 9, 1-iododecane 10, *N,N*-dihexylamine 11, and *N,N,N*-trioctylamine 12 (Schemes 1, 2). *O*-Toluidine is carcinogenic and therefore was not used in synthetic experiments.

**TABLE 1:** Biological origin of samples of interest.

Entry^†^	AIMS Sample Code	Biological origin	QCL Sample Number	Fraction
4	20608	*Lendenfeldia* sp.	SN00760947	Crude extract
6	26051	*Ircinia gigantea*	SN00731005	Crude extract
10	19033	*Dysidea herbacea*	SN00733110	Crude extract
13	20608	*Lendenfeldia* sp.	SN00760956	75% MeOH eluent
14	20608	*Lendenfeldia* sp.	SN00760958	100% MeOH eluent
16	24307	Class Demospongiae^‡^	SN00730755	75% MeOH eluent

*^†^Corresponds to Entry Number in Table 1 above. ^‡^More detailed taxonomic classification not available.*

Compounds 1a, 1b, and 2a were prepared using 1-bromooctane 6 to alkylate *m*-toluidine 7, *p*-toluidine 8, benzyl amine 9, respectively (Scheme 1), giving three compounds based on the active component of AIMS sample 20608. Compound 3, the active component of AIMS sample 19033, was prepared by reacting 1-iododecane 10 with *N,N*-dihexylamine 11 (Scheme 2), while *N,N,N*-trioctylamine hydrochloride 13 was prepared from the free amine 12 as a simple and readily accessible analog of the natural product structures 4 and 5 that had been isolated from AIMS sample 26051.

**Scheme 3:**
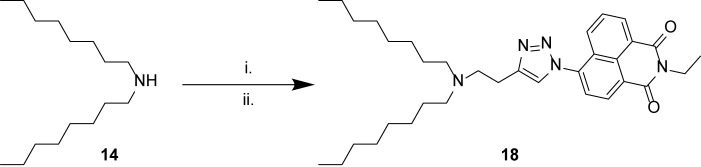
Synthesis of naphthalimide derivative 18. (i) 4-bromo-1-butyne 15, K2CO3, KI, MeCN, 60°C, overnight, 2%; (ii) 6-azido-2-ethyl-1*H*-benzo[*de*]isoquinoline-1,3(2*H*)-dione 17, CuSO4, sodium ascorbate, tBuOH/H2O, 60°C, overnight, 1%.

Finally, we sought to combine the tertiary amine structures elucidated in this study with a triazolyl naphthalimide pendant, to result in a fluorescent label compound for visualization of fluorescent compounds in the cells. Thus *N,N*-dioctylamine 14 was alkylated with 4-bromo-1-butyne 15, and the resulting alkyne product 16 “clicked” with 6-azido-2-ethyl-1*H*-benzo[*de*]isoquinoline-1,3(2*H*)-dione 17 (Yu et al., 2013) to afford the naphthalimide derivative 18 (Scheme 3).

While the yields of many synthetic steps were low, sufficient quantities of material were nonetheless isolated to enable characterization, and biological evaluation, so the synthetic reactions were not further optimized.

### Structural Comparison of Synthetic Compounds to Natural Products

**FIGURE 1:**
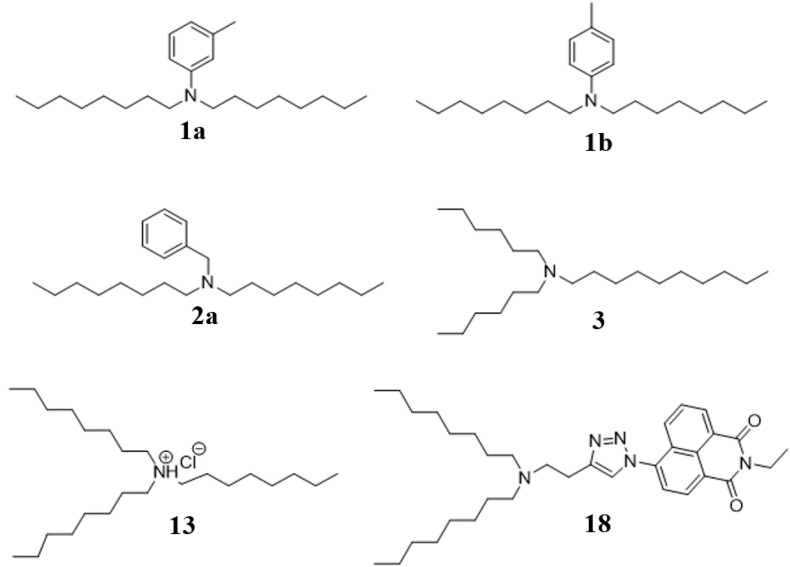
Synthesis structures of bioactive amine natural products identified as new compounds in this study.

Synthetic compounds (Figure 1) were investigated using mass spectrometry (MS/MS and accurate mass) and analysis of biological activity. Comparing the major ions in the mass spectra of synthetic 1a and 1b (Supplementary Table S3), 2a (Supplementary Table S4), 3 (Supplementary Table S5), and 13 (Supplementary Table S6) with the natural products shows good correlation that we have previously reported (Dinarvand and Peter Spain, 2020). Some minor differences are apparent, which most likely arise due to differences in the amounts of material analyzed (which are significantly greater for the synthesized products), and differences in the instrumentation used.

### Antibacterial Activity and Toxicity of Synthetic Compounds

Synthetic compounds were assessed against MRSA, *P. aeruginosa*, *uropathogenic E. coli*, and *M. tuberculosis* for antimicrobial and selectivity’s activity. Compound selectivity is one of the key properties that successful drugs need to deliberate simultaneously so as to determine how a compound can differentially bind to only the target of interest with high affinity (i.e., high activity) while binding to other proteins with low affinities (Liu and Ning, 2017). However, the compound selectivity and mechanism in the drug development process have to be considered more.

Interestingly, all synthetic derivatives compounds showed similar MICs against MRSA (the organism against which the original natural product screening assays had been conducted), typically around 12.5 μM. The simple amine salt 13 proved the most effective of the synthetic compounds against *M. tuberculosis* with an MIC of 0.02 μM and showed moderate inhibitory activity against the other bacteria. Compound 3 displayed broad activity, with low MICs against *P. aeruginosa* (MIC 3.1 μM), *E. coli* (6.2 μM), and *M. tuberculosis* (3.1 μM). The naphthalimide derivative 18 displayed good selective activity against *E. coli* (MIC 1.5 μM) (Table 2).

**TABLE 2:** Anti-bacterial activity of synthetic compounds against MRSA, *P. aeruginosa*, *E. coli*, and *M. tuberculosis*.

Synthetic compound	MIC (μM)^†,‡^			
	MRSA	*M. tuberculosis*	*E. coli*	*P. aeruginosa*
1a	12.5 ± 1.0	1.5 ± 3.0	12.5 ± 5.0	6.2 ± 9.0
1b	100 ± 5.4	25 ± 1.0	100 ± 1.0	100 ± 6.5
2a	12.5 ± 0.7	1.5 ± 1.0	50 ± 1.0	50 ± 1.0
3	12.5 ± 1.0	3.1 ± 0.7	6.2 ± 1.0	3.1 ± 5.0
13	12.5 ± 2.7	0.02 ± 1.1	25 ± 2.0	25 ± 4.0
18	12.5 ± 2.0	12.5 ± 1.0	1.5 ± 4.0	12.5 ± 0.8

*^†^Samples were diluted from 100 to 0.0002 μM and incubated with bacteria (starting concentration OD600 = 0.001) under optimum assay conditions as described in the Experimental section. ^‡^Values represent averages from three independent repeats.*

The potential toxicity of the synthetic compounds was also evaluated, against A549, THP-1, HepG2 and HEK 293 cell lines (Table 3). None of the synthetic compounds showed significant toxicity against HepG2 or A549 cells. Synthetic compounds 1a, 2a, 3, 13 and **18** all showed some toxicity against THP1 and/or HEK 293 cells, with minimum toxicity concentrations (MTC) as low as 3.1 μM. Compound 1b showed low toxicity against all four of these cell lines, but also low activity (Table 3). Compound 1a showed only mild effects on all cell lines tested (MTC 50–100 μM), while it also displayed a broad antibacterial profile, suggesting this compound may be a candidate for further investigation.

**TABLE 3:** Toxicity of synthetic compounds to cell lines.

Synthetic samples	Minimum toxicity concentration of drug (MTC μM)^†,‡,§^			
	A549	THP1	HepG2	HEK 293
1a	>100 ± 4.1	50 ± 2.0	>100 ± 3.0	50 ± 0.9
1b	>100 ± 3.8	>100 ± 2.0	>100 ± 0.9	>100 ± 6.0
2a	>100 ± 1.0	6.5 ± 0.8	>100 ± 0.8	50 ± 4.0
3	>100 ± 2.0	50 ± 5.0	>100 ± 1.0	25 ± 2.0
13	50 ± 1.4	3.1 ± 0.7	>100 ± 5.2	6.5 ± 3.0
18	>100 ± 6.0	>100 ± 9.0	>100 ± 5.0	3.1 ± 8.2

*^†^Cells seeded at a concentration of 2 × 105 cells/well; samples diluted 100 to 0.0002 μM. ^‡^Cells incubated with sample under humidified incubation of 37°C with 5% CO_2_. ^§^Values represent averages of three independent repeats.*

Comparing data from the antimicrobial activity and cytotoxicity assays shows that the active concentration ranges for these synthetic compounds against bacteria are substantially lower than active concentration ranges against the mammalian cells tested, particularly HepG2 and A549 cells. THP1 cells and the primary cells (data not shown) appeared more sensitive to these compounds. Cross-referencing the biological activity and toxicity data for these compounds suggest that they have some potential for further development.

Compound 13 exhibited strong inhibitory activity against all resistant strains with MICs as low as 0.24 μm; Compound 1a showed moderate and variable activity against all assay strains (MIC 6.7–20.0 μm) and 18 showed similar activity against all resistant strains with an MIC of 2.2 μm. In this study, MRSA and *M. tuberculosis* was chosen as a candidate for future investigation.

### Checkerboard Assays

**FIGURE 2:**
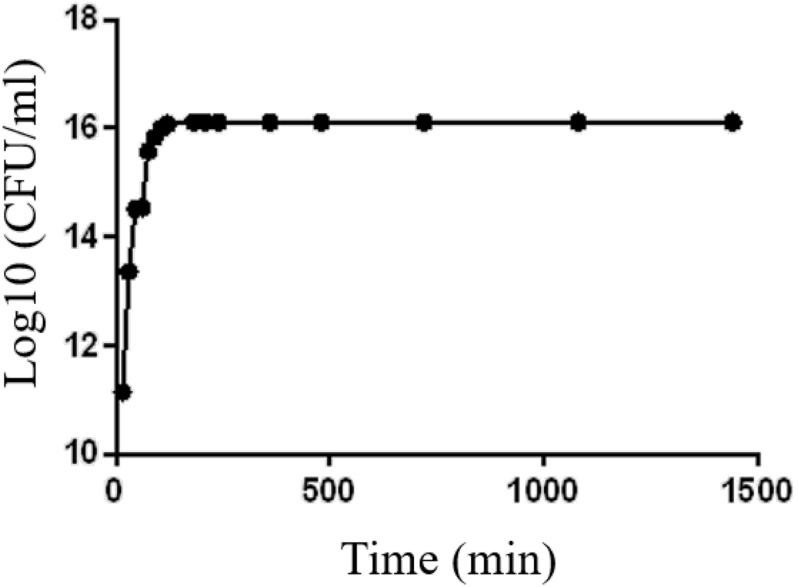
Growth curve of MRSA grown at 37°C in LB for 24 h.

**FIGURE 3:**
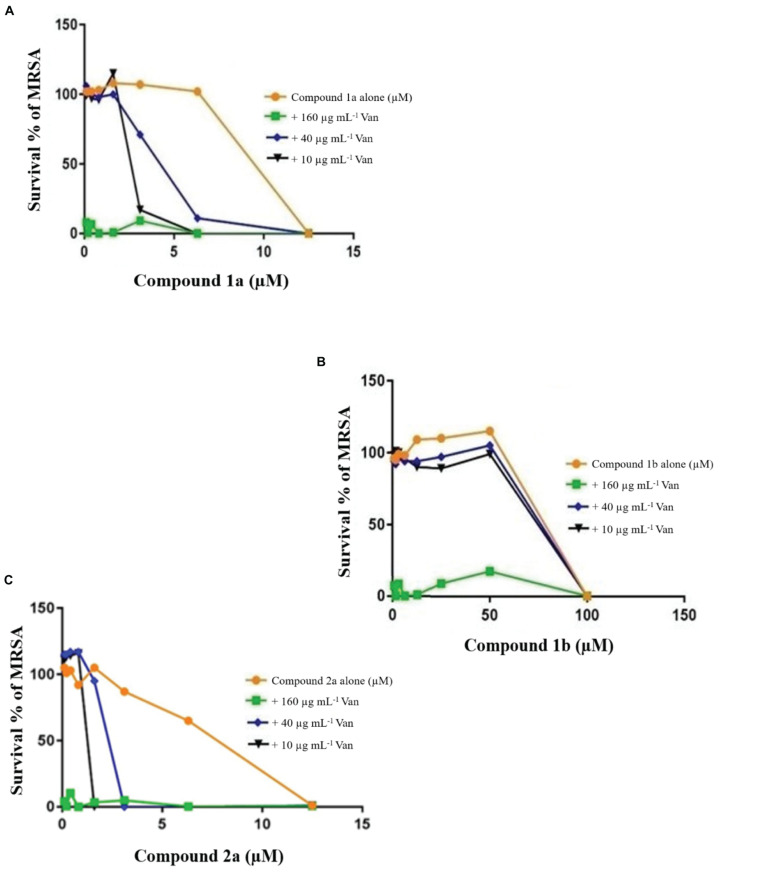
Checkerboard study between vancomycin 1a **(A)**, 1b **(B)**, and 2a **(C)**. MRSA was incubated with varying concentrations of vancomycin (Van) together with dilutions of the synthetic drugs 2a **(A)**, 1a **(B)**, and 1b **(C)**. Graph displays the mean survival of triplicate samples and represents data from three independent repeats.

**FIGURE 4:**
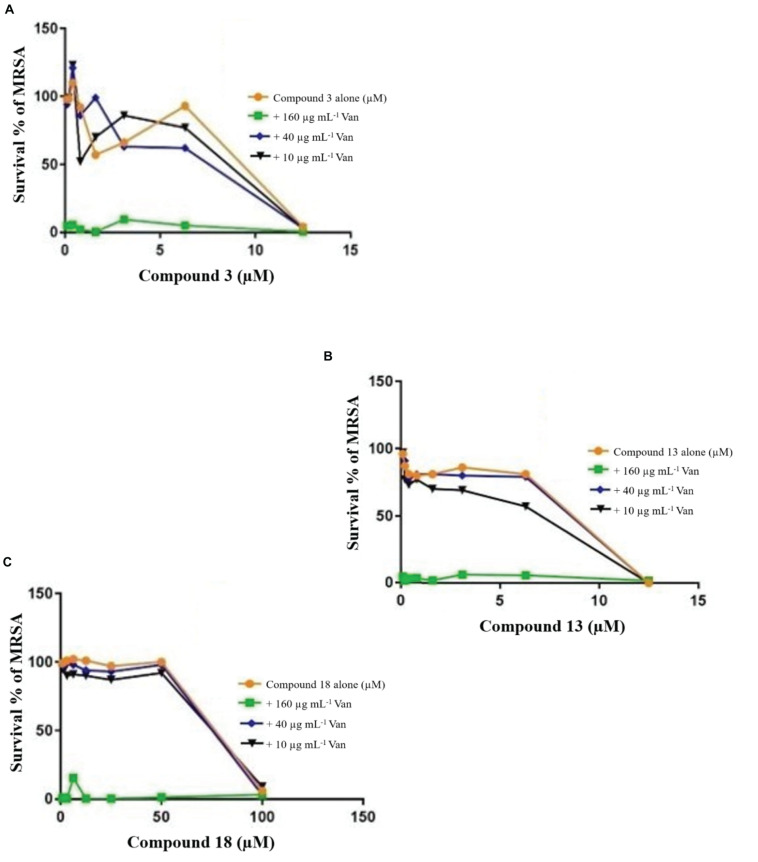
Checkerboard study between vancomycin and 3 **(A)**, 13 **(B)**, and 18 **(C)**. MRSA was incubated with varying concentrations of vancomycin (Van) together with dilutions of the synthetic drugs 3 **(A)**, 13 **(B)**, and 18 **(C)**. Graph displays the mean survival of triplicate samples and represents data from three independent repeats.

**FIGURE 5:**
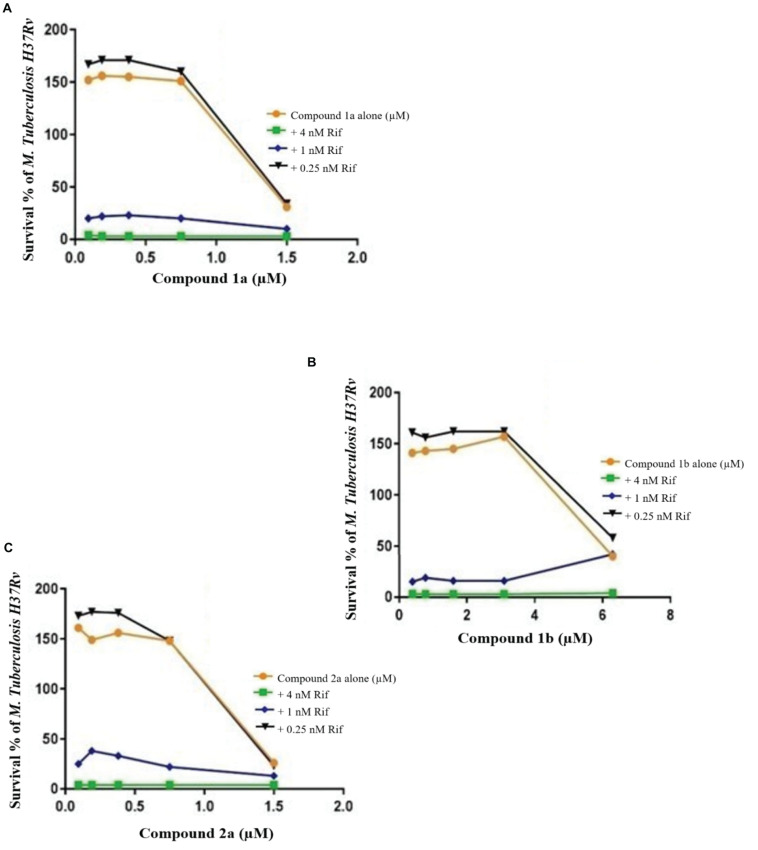
Checkerboard study between rifampicin and 1a **(A)**, 1a **(B)**, and 2a **(C)**. *TB. H37Rv* was incubated with varying concentrations of rifampicin (Rif) together with dilutions of the synthetic drugs 1a **(A)**, 2a **(B)**, and 1b **(C)**. Graph displays the mean survival of triplicate samples and represents data from three independent repeats.

**FIGURE 6:**
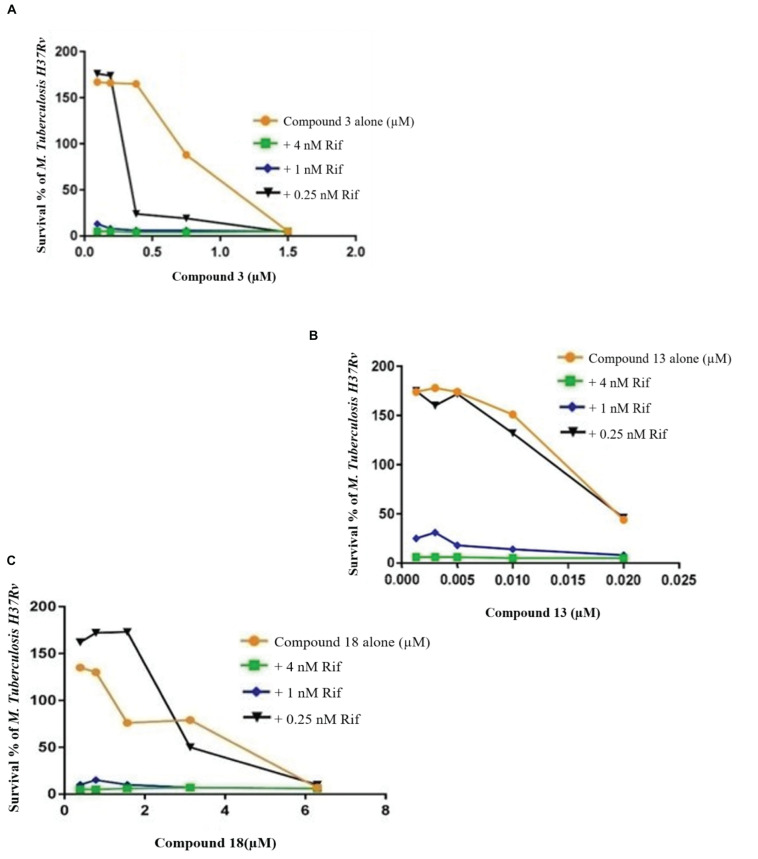
Checkerboard study between rifampicin and 3 **(A)**, 13 **(B)**, and 18 **(C)**. *TB. H37Rv* was incubated with varying concentrations of rifampicin (Rif) together with dilutions of the synthetic drugs 3 **(A)**, 13 **(B)**, and 18 **(C)**. Graph displays the mean survival of triplicate samples and represents data from three independent repeats.

Growth curves for the MRSA confirmed that growth was well supported in LB medium and log phase (exponential) growth period estimated between 30 and 60 min (Figure 2) and TB in Middlebrook 7H9 broth between 1 and 2 days. Previously, the combination of known drugs against MRSA and *M. tuberculosis* was reported (Maltempe et al., 2017; Bakthavatchalam et al., 2019). In this study, the CA applied in triplicate for MRSA and *M. tuberculosis* to assess the bactericidal activity of synthesized samples in combination with known drugs (Figures 3–6). The results from this work showed that certain combination regimens displayed improved bactericidal activities. All treatments with combinations of commercial antimicrobial agents at suboptimal MIC were effective to some extent against the test pathogens, however effects were not consistent. Vancomycin plus compounds 2a and 1a displayed bactericidal activity against MRSA (Figure 3). On the other hand, synthetic samples in combination with rifampicin were typically not effective against *M. tuberculosis*, except for compounds 3 and 18 that showed synergistic or bactericidal effects (Figure 6). Thus, some of our newly discovered antimicrobial agents have the potential to work in combination with existing drugs to combat drug resistant bacteria, a result that is in accordance with previous studies.

The FIC index of vancomycin and rifampicin with four and five synthesized compounds for each of the 2 tested strains, ranged between 1 to 1.2, respectively (Table 4), meaning that under the classical conditions of this CA, antagonism (no interaction) was demonstrated between these two combinations. Synergistic effects were observed with 2a and 1a when 10 μg mL^–1^ vancomycin was combined with 1.6 μM 2a and 3.1 μM 1a (Figure 4), which showed FIC = 0.328 and 0.45, respectively.

**TABLE 4:** Summary of the checkerboard analysis between vancomycin and synthetic compounds.

Structure name	Compound alone MIC50 (μM)	Compound MIC50 for MRSA in presence of Van (10 μg mL^–1^)	FIC	Drug–drug interactions
1a	12.5	3.1	0.45	Synergy
1b	100	100	1.2	Indifference
2a	12.5	1.5	0.33	Synergy
3	12.5	12.5	1.2	Indifference
13	12.5	6.25	1.2	Indifference
18	12.5	12.5	1.2	Indifference

Compound 3 in concentration of 1.5 μM shown synergism effect on *M. tuberculosis* H37Ra, in our study when combine with 0.25 nM rifampicin (Figure 6). The results of the “dynamic checkerboard,” which quantifies bacterial growth when drugs are used in combination, showed that compounds alone reduced the bacteria by >1 to 2 log10 CFU mL^–1^, while in combination with known drugs bacterial numbers were reduced at maximum by >2 to 3 log10 CFU mL^–1^ (Table 5).

**TABLE 5:** Summary of the checkerboard study between rifampicin and synthetic compounds.

Structure name	Compound alone MIC50 (μM) for *M. tuberculosis*	Compound MIC50 for *M. tuberculosis* in presence of Rif (0.25 nM)	FIC	Drug–drug interactions
1a	1.5	1.5	1.1	Indifference
1b	6.25	6.25	1.1	Indifference
2a	1.5	1.5	1.1	Indifference
3	1.5	0.38	0.4	Synergy
13	0.02	0.02	1.1	Indifference
18	6.25	3.13	1	Indifference

### Time-Kill Curves

An additional important step in the drug development process is evaluation of pharmacodynamics *in vitro*, a necessary step before animal experiments. One important assay is use of time-kill curves, which monitor antibacterial effect on bacterial growth and death over times at a wide range of antimicrobial concentration. In this study, we obtain and compare *in vitro* pharmacodynamic parameters of synthetic compounds and commercial drugs, opening up avenues into understanding the effects of different concentration of synthetic compounds alone and in combinations with commercial drugs on resistant bacteria that describes the relationship between the concentration of synthetic compounds and commercial drugs with bacterial growth rate.

**FIGURE 7:**
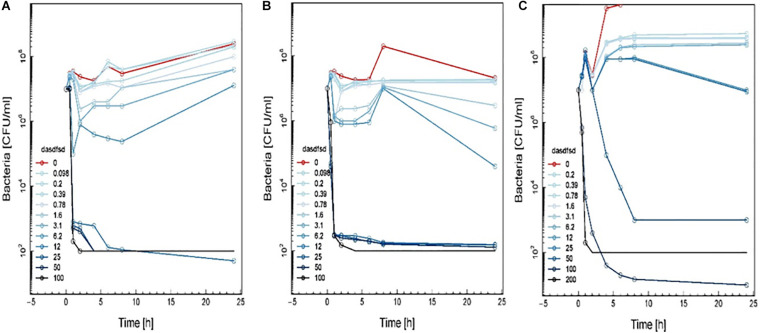
Time-kill curve study of 2a **(A)**, 1a **(B)**, and 1b **(C)** versus MRSA in log-phase at drug exposure. The curve displays the mean CFU for duplicate samples and represents data from two independent experiments. Twelve doubling dilutions are plotted, the highest concentration (*black line*) corresponds to 16 × MIC. Growth in absence of antimicrobial is drawn in *red*. The compound was added at time point 0 and monitored until 24 h. The limit of detection in the assay was 100 CFU mL^–1^.

The time-kill assay we developed worked well for different synthetic compounds, including commercial drugs. The results showed the MIC and MBC were time-dependent and should be read within 18 to 24 h and (Table 6). Time-kill curves for 1a, 1b, and 2a using the MRSA (≤ MIC ≥) are shown in Figure 7. Concentrations of up to 12.5 μM of these synthetic compounds induced a bactericidal effect, but the onset of the bactericidal activity was dependent on the concentration of the antimicrobial and differed between synthetic compounds. MRSA were killed to below the limit of detection (100 CFU/mL) at the highest antimicrobial concentration (16-fold MIC). MRSA bactericidal activity of synthetic compounds at concentration 12.5 to 50 μM decreased during the 4 to 6 h of the assay.

**TABLE 6:** Summary of MIC and MBC.

Structure name	Compound (μm/mL)			
	MIC for MRSA	MBC for MRSA	MIC for *TB. H37Rv*	MBC for *TB. H37Rv*
1a	12.5	25	1.5	25
1b	100	200	6.25	100
2a	12.5	100	1.5	25
3	12.5	12.5	1.5	6.25
13	12.5	100	0.02	0.39
18	12.5	12.5	6.25	50

**FIGURE 8:**
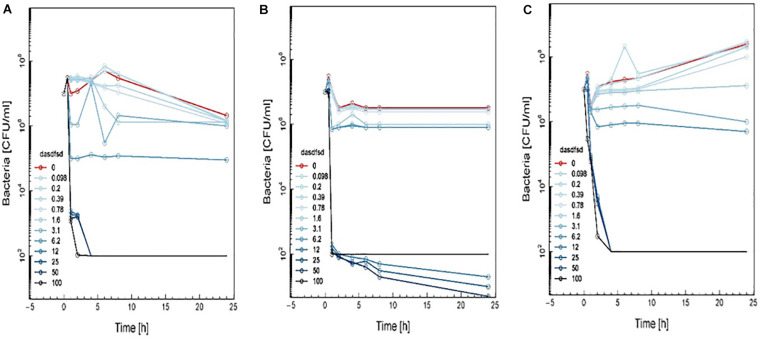
Time-kill curve study of 3 **(A)**, 13 **(B)**, and 18 **(C)** versus MRSA in log-phase at drug exposure. The curve displays the mean CFU for duplicate samples and represents data from two independent experiments. Twelve doubling dilutions are plotted, the highest concentration (*black line*) corresponds to 16 × MIC. Growth in absence of antimicrobial is drawn in *red*. The compound was added at time point 0 and monitored until 24 h. The limit of detection in the assay was 100 CFU mL^–1^.

**FIGURE 9:**
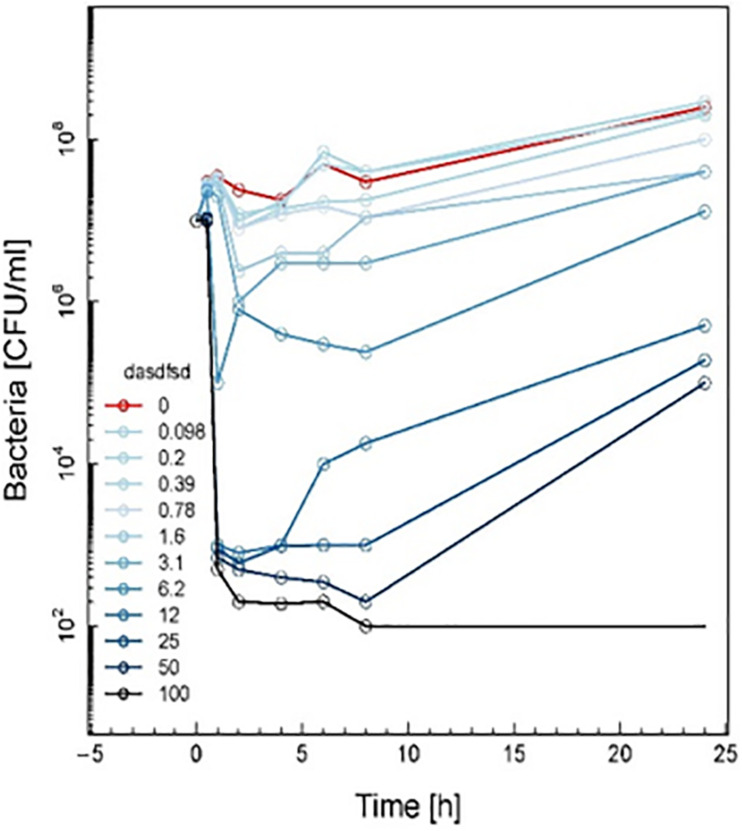
Time-kill curve study of vancomycin versus MRSA in log-phase at drug exposure. The curve displays the mean CFU for duplicate samples and represents data from two independent experiments. Twelve doubling dilutions are plotted, the highest concentration (*black line*) corresponds to 16 × MIC. Growth in absence of antimicrobial is drawn in *red*. The compound was added at time point 0 and monitored until 24 h. The limit of detection in the assay was 100 CFU mL^–1^.

Time-kill curves for three additional compounds (3, 13, and 18) were also made (Figure 8). Similar to the effect of 2a, 1a, and 1b (Figure 7) exhibited rapid killing during the first 2 h of the assay for concentrations above MIC. Compounds up to 12.5 μM exhibited a bactericidal effect against log-phase MRSA but compound 1b at 100 μM concentration rapidly reduced growth by 1 h post-treatment, followed by inhibition of growth up to 6 h (Figure 8). For vancomycin (Figure 9), concentrations up to 50 μg mL^–1^ did not have an antibacterial effect compared to untreated bacteria. At this concentration, initial killing to 102–104 CFU mL^–1^ until 8 to 12 h was observed, which was followed by regrowth to 106 CFU mL^–1^ after 24h. For vancomycin ≥50 μg mL^–1^, persistent killing without re-growth was observed and a bactericidal effect was obtained for vancomycin 100 μg mL^–1^ at 24 h.

Based on these results, the effect of compounds 18 and 3 was slightly more rapid killing at 12.5 μg mL^–1^ which is the MIC50, while for compound 13 killing was observed at the highest concentration (>100 μg mL^–1^) as compared to inhibition concentration. Since compounds 3 and 13 are related to the same class II compounds but display different behaviors in the killing time study, this could be explained by each having a different mechanism of action. Interestingly, the checkerboard and TKS illustrated decreases in the viable cell counts for these same synthetic compounds over 4 or 6 h, but regrowth was noted at 24 h (Figures 3–8). The *in vitro* pharmacodynamic parameters suggest that there is a continuous gradient from bacteriostatic to bactericidal effects and that compound 1b might fall in between these two categories.

**FIGURE 10:**
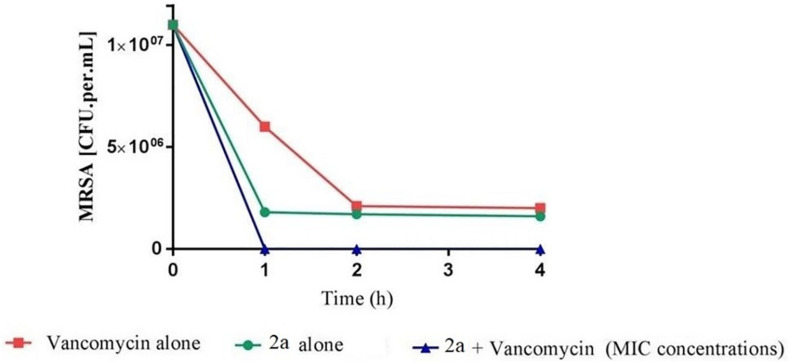
Time-kill curve study of combinations of vancomycin and 2a versus MRSA at drug exposure in MIC concentrations. The curve displays the mean CFU for duplicate samples and represents data from two independent experiments. Twelve doubling dilutions are plotted, the highest concentration (*black line*) corresponds to 16 × MIC. Growth in absence of antimicrobial is drawn in *red*. The compound was added at time point 0 and monitored until 24 h. The limit of detection in the assay was 100 CFU mL^–1^.

Sub-inhibitory combinations of compounds 2a, 1a, 1b, 3, 13, 18, and vancomycin substantially inhibited growth of MRSA and displayed an increased antibacterial effect compared to the effect of each agent alone. In combination, compound 2a at 1.5 to 0.1 μM and vancomycin (50 μg mL^–1^) resulted in protracted growth of MRSA but net-growth up to 102 CFU mL^–1^ at 24 h. The addition of sub-inhibitory vancomycin at 50 μg mL^–1^, which displayed no effect alone, and 12.5 μM of compound 1b substantially reduced regrowth of the MRSA that was observed with compound 2a at 25 μM alone. From these combinations of vancomycin at 50 μg mL^–1^ and compound 2a at 12.5 μM a bactericidal effect was observed in 1 h (Figure 10). Hence, a favorable interaction between vancomycin and all compounds was observed. The *in vitro* pharmacodynamic parameters can provide relative comparisons across different synthesized compounds and current antimicrobials which can be extremely valuable in preclinical studies but furthermore, pharmacokinetic effects need to be study. A novel synthesized compound can be categorized and compared to mechanistically well-understood antibiotics.

The activities of rifampicin alone and in combine with six synthesized compounds against *M. tuberculosiss* were determined. Results showed that, while significant reduction in bacterial viability was observed at day 6 following compounds 1a, 1b, 2a, and 3 treatments, exposure to rifampicin and compound 3, 13, and 18 achieved comparable killing effects at day 7 of treatment (data not shown).

## Conclusion

Vancomycin and rifampicin remain the main agents for the treatment of invasive MRSA and *M. tuberculosis* diseases, respectively. Yet the number of vancomycin-resistant *S. aureus* (VRSA) and rifampicin-resistant *M. tuberculosis* strains is on the rise. The emergence of antibiotic resistance brings a need for novel, effective antibacterial agents that are resistant to antimicrobial resistance.

The pharmacodynamic parameters can be applied for the evaluation of new antimicrobials and to study the effects of combining antimicrobials against MRSA and *M. tuberculosis*. Synthetic compounds based on the natural product structures (Dinarvand and Peter Spain, 2020) were prepared to validate and expand these findings. Synthetic compounds 1a, 1b, 2a, 3, and 13 showed promising bioactivity/toxicity profiles. Naphthalimide derivative 18 was prepared as a hybrid of the amines uncovered in this study.

The compounds uncovered in this study add to the growing arsenal of antimicrobial agents from the sea (Hughes and Fenical, 2010; Indraningrat et al., 2016) and offer interesting new avenues for further investigation in the quest for new, effective agents to combat the growing scourge of multidrug resistant bacteria.

An additional important step in the drug development process is evaluation of pharmacodynamics, a necessary step before animal experiments. The CA and sTKS are two important assays to monitor antibacterial effect on bacterial growth and death overtime at a wide range of antimicrobial concentration. All treatments with combinations of commercial antimicrobial agents at suboptimal MIC were effective to some extent against the tested pathogens, however, effects were not consistent. Our results suggest that rates of synergy for vancomycin and rifampicin with synthetized compounds for the test organisms in this study are comparable, although antagonism was observed more frequently with the latter.

In conclusion, that this could be the case for Compound 1a, 2a and 3, which were found to be strongly bactericidal and concentration dependent. More extensive work with a variety of combinations is needed to confirm this impression, but we believe that such work is warranted on the basis of the results presented in this work.

## Data Availability Statement

The raw data supporting the conclusions of this article will be made available by the authors, without undue reservation.

## Author Contributions

MD conceived, designed the experiments, and wrote the manuscript. MD and MS performed the experiments. MD, MS, and FV analyzed the data. FV oversighted statistical modelling and revised the manuscript. All authors contributed to the article and approved the submitted version.

## Conflict of Interest

The authors declare that the research was conducted in the absence of any commercial or financial relationships that could be construed as a potential conflict of interest.

## Abbreviations

A549: human alveolar epithelial cells
AIMS: Australian Institute for Marine Science
DMEM: Dulbecco’s Modified Eagle’s medium
HEK293: human embryonic kidney cells 293
Hep-G2: human hepatocellular carcinoma cells
HPLC: high-performance liquid chromatography
HTS: high-throughput screening
MDCK: Madin-Darby canine kidney epithelial cells
MIC: minimum inhibitory concentration
MRSA: methicillin resistant *Staphylococcus aureus*
MS: mass spectrometry
MS/MS: tandem mass spectrometry
MTC: minimum toxic concentration
NMR: nuclear magnetic resonance
RPMI: Roswell Park Memorial Institute Medium
THP-1: human leukemia cells
VRSA: vancomycin-resistant *Staphylococcus aureus*.
