# Supplementary material for: Pharmacodynamic Functions of Synthetic Derivatives for Treatment of Methicillin-Resistant Staphylococcus aureus (MRSA) and Mycobacterium tuberculosis
Source: Front Microbiol. 2020 Nov 27;11:551189. doi: 10.3389/fmicb.2020.551189 (PMC7729195; doi:10.3389/fmicb.2020.551189)
Supplement: Supplementary file 1 [file Data_Sheet_1.docx]

**TABLE S1. Determination of MIC values for selected compounds against MRSA.** MRSA was incubated with samples as described in the Experimental section.

| **Entry** | **AIMS Sample Code** | **Phylum** | **QCL Sample Number** | **Fraction** | **MRSA MIC**  **(µg mL^−1^)** |
| --- | --- | --- | --- | --- | --- |
| **1** | 22565 | Porifera | SN00739718 | Crude Extract | 31.3 ± 1.5 |
| **2** | 24606 | Porifera | SN00732793 | MeOH Eluent | 125.0 ± 4.0 |
| **3** | 24307 | Porifera | SN00730771 | MeOH Eluent | 250.0 ± 2.0 |
| **4^†^** | 20608 | Porifera | SN00760947 | Crude Extract | 31.3 ± 1.0 |
| **5** | 25641 | Porifera | SN00731867 | Crude Extract | 250.0 ± 2.5 |
| **6^†^** | 26051 | Porifera | SN00731005 | Crude Extract | 62.5 ± 4.0 |
| **7** | 25642 | Porifera | SN00731868 | Crude Extract | 125.0 ± 1.6 |
| **8** | 24132 | Porifera | SN00734102 | Crude Extract | 125.0 ± 0.6 |
| **9** | 19033 | Porifera | SN00733107 | MeOH Eluent | 62.5 ± 0.1 |
| **10^†^** | 19033 | Porifera | SN00733110 | Crude Extract | 31.3 ± 1.0 |
| **11** | 19039 | Porifera | SN00733134 | Crude Extract | 125.0 ± 2.0 |
| **12** | 19039 | Porifera | SN00733131 | MeOH Eluent | 250.0 ± 0.2 |
| **13^†^** | 20608 | Porifera | SN00760956 | 75% MeOH Eluent | 31.3 ± 0.5 |
| **14^†^** | 20608 | Porifera | SN00760958 | MeOH Eluent | 62.5 ± 3.0 |
| **15** | 25691 | Porifera | SN00732374 | Crude Extract | 125.0 ± 1.1 |
| **16^†^** | 24307 | Porifera | SN00730755 | 75% MeOH Eluent | 31.3 ± 0.1 |
| **17** | 24307 | Porifera | SN00730707 | Crude Extract | 500.0 ± 10 |
| **18** | 25663 | Chordata | SN00732222 | Crude Extract | 15.6 ± 3.0 |
| **19** | 25663 | Chordata | SN00732228 | 75% MeOH Eluent | 125.0 ± 1.0 |
| **20** | 26104 | Porifera | SN00734298 | Crude Extract | 62.5 ± 2.0 |
| **21** | 25658 | Porifera | SN00732162 | Crude Extract | 125.0 ± 1.4 |
| **22** | 25658 | Porifera | SN00732159 | MeOH Eluent | 500.0 ± 1.4 |
| **23** | 24348 | Echinodermata | SN00739901 | 30% MeOH Eluent | 500.0 ± 4.0 |

^†^ Samples with promising biological activity that were subjected to further investigation.
